# Inflammatory Cytokines in Diabetic Nephropathy

**DOI:** 10.1155/2015/948417

**Published:** 2015-02-15

**Authors:** Javier Donate-Correa, Ernesto Martín-Núñez, Mercedes Muros-de-Fuentes, Carmen Mora-Fernández, Juan F. Navarro-González

**Affiliations:** ^1^Research Unit, University Hospital Nuestra Señora de Candelaria, 38010 Santa Cruz de Tenerife, Spain; ^2^Clinical Biochemistry Service, University Hospital Nuestra Señora de Candelaria, 38010 Santa Cruz de Tenerife, Spain; ^3^Nephrology Service, University Hospital Nuestra Señora de Candelaria, 38010 Santa Cruz de Tenerife, Spain

## Abstract

Probably, the most paradigmatic example of diabetic complication is diabetic nephropathy, which is the largest single cause of end-stage renal disease and a medical catastrophe of worldwide dimensions. Metabolic and hemodynamic alterations have been considered as the classical factors involved in the development of renal injury in patients with diabetes mellitus. However, the exact pathogenic mechanisms and the molecular events of diabetic nephropathy remain incompletely understood. Nowadays, there are convincing data that relate the diabetes inflammatory component with the development of renal disease. This review is focused on the inflammatory processes that develop diabetic nephropathy and on the new therapeutic approaches with anti-inflammatory effects for the treatment of chronic kidney disease in the setting of diabetic nephropathy.

## 1. Introduction

Diabetes-related complications represent one of the most important health problems worldwide with dire projections. One of the most important medical concerns of the diabetes epidemic is diabetic nephropathy (DN). Approximately one-third of all diabetic patients are affected by DN [1], which produces significant social and economic burdens [2] and constitutes the most frequent cause of end-stage renal disease (ESRD) [3, 4]. In addition, renal involvement is a major cause of morbidity and mortality in the diabetic population, with this epidemic being likely to drive us to previously unforeseen rates of vascular target organ complications.

The concept of the underlying pathophysiologic processes leading to DN has evolved tremendously. In the classical view, renal injury in these patients is explained by metabolic and hemodynamic alterations, which increase systemic and intraglomerular pressure, and by the modification of molecules under hyperglycemic conditions. This view has evolved to a much more complex scenario, where the pathogenesis of DN appears as multifactorial, with both genetic and environmental factors triggering a complex series of pathophysiological events [5, 6]. Intensive research in recent years on the aetiology of DN at the cellular and molecular level has given rise to inflammation as a key pathophysiological mechanism. Understanding the key features of inflammatory mechanisms involved in the development and progression of diabetic kidney injury will enable the identification of new potential targets and facilitate the design of innovative anti-inflammatory therapeutic strategies.

This review is focused on the pathogenesis of DN associated with the inflammatory process. We focus on proinflammatory molecules and pathways related to the development and progression of renal injury, discuss the potential clinical use of inflammatory markers as predictors of DN, and comment upon potential new strategies to treat DN using agents that target inflammatory pathways.

## 2. Inflammation

There now are convincing data that diabetes includes an inflammatory component that is thought to be related to diabetic complications. Our understanding of the role of this component is still restricted to specific molecules and single pathways, so our understanding of the highly complex and diverse molecular interactions that occur in the kidneys of patients with DN is very superficial.

Diabetes mellitus is associated with a myriad of deviations from normal homeostasis which includes hemodynamic abnormalities (resulting from systemic and intraglomerular hypertension, altered shear stress, and mechanical strain), metabolic derangements (hyperglycemia, formation of advanced glycation end products, and hyperlipidemia), and increased synthesis of hormones such as angiotensin II. Additionally, an increasing number of studies suggest that oxidative stress, inflammation, and fibrosis appear to be the key links in the progression of DN. Oxidative stress is the initial part of DN and activates a variety of pathological pathways in virtually all types of kidney cells (endothelial, mesangial, epithelial, tubular cells, and podocytes). However, fibrosis is the most fundamental and prominent feature of DN and inflammation appears to be the central role [7] in the onset and progression of kidney fibrosis if uncontrolled.

Plasma concentrations of inflammatory molecules, including proinflammatory cytokines, are elevated in diabetic patients [8–10]. Recent studies have shown that concentrations of these substances increase as nephropathy progresses [11, 12] and that these inflammatory molecules are independently related to urinary albumin excretion (UAE) [12, 13] presenting a direct association with clinical markers of glomerular and tubulointerstitial damage. The extent of inflammatory cell accumulation in the kidney is closely associated with DN [14–18]. Indeed, inhibition of inflammatory cell recruitment into the kidney has been shown to be protective in experimental diabetic nephropathy [19, 20]. Together, these results suggest that inflammation may be a pathogenic factor for the development and progression of DN [21]. Proinflammatory and fibrogenic cytokines synthesized and secreted by these cells in the local microenvironment directly damage kidney architecture and subsequently trigger the epithelial-to-mesenchymal transition process [22], resulting in extracellular matrix accumulation. Not only the synthesis of proinflammatory cytokines, but also the expression of chemoattractant cytokines and adhesion molecules are upregulated in animal and patients kidney cells with diabetes. These molecules are key mediators of renal injury by virtue of their ability to attract circulating white blood cells (monocytes, neutrophils, and lymphocytes) and facilitate transmigration of these cells into renal tissue. These infiltrating cells are also a source of cytokines and other mediators that contribute to the development and progression of renal injury, as well as to amplification and perpetuation of the inflammatory reaction.

Immunologic and inflammatory mechanisms play a significant role in development and progression of DN [23, 24] with recruitment and activation of innate immune cells and elaboration of proinflammatory cytokines. Thereby, macrophages and T-lymphocytes, which are prominent in diabetic glomeruli [25, 26], as well as different molecules, such as chemokines [27, 28], adhesion molecules [20, 29], growth factors [30–33], nuclear factors [34, 35], and cytokines [21], have been implicated in diverse pathogenic pathways related to DN.

## 3. Inflammatory Cytokines in the Pathophysiology of Diabetic Nephropathy

Cytokines are a group of pharmacologically active, low molecular weight polypeptides with autocrine, paracrine, and juxtacrine effects which, in a coordinated manner, regulate inflammatory and immune responses with the participation of different cytokine-associated signalling pathways. Cytokines are produced throughout the body by cells of varied embryological origin and, in addition to their immune response regulatory role, exert important pleiotropic actions as cardinal effectors of injury [36]. At present time, it is recognized that chronic low-grade inflammation and activation of the innate immune system are closely involved in the pathogenesis of diabetes mellitus [37–39]. Plasma concentrations of diverse inflammatory parameters are elevated in diabetic patients [8–10, 40, 41] being strong predictors of the development of this disease [42–44].

A potential participation of inflammatory cytokines in the pathogenesis of DN was suggested for the first time in 1991 by Hasegawa et al. [45]. In this work, authors demonstrated that peritoneal macrophages cultured with glomerular basement membranes from diabetic rats produced significantly higher amounts of the inflammatory cytokines tumor necrosis factor-alpha (TNF-*α*) and interleukin- (IL-) 1 than those cultured with glomerular basement membranes from normal rats. Subsequent studies demonstrated that, in the kidney, both blood-borne cells (mainly monocytes and macrophages) and diverse intrinsic renal cells (endothelial, mesangial, dendritic, and tubular epithelial cells) are able to synthesize inflammatory cytokines [46–50]. Furthermore, the levels of these substances increase as nephropathy progresses [11, 12, 48], with an independent relationship between inflammatory parameters and urinary albumin excretion (UAE) [12, 13] suggesting a role of this substances in the pathogenesis of DN [13, 49, 50].

Inflammatory cytokines involved in the pathogenesis of diabetes play a significant role in the development and progression of several renal disorders [51], including DN [13, 45, 50]. The renal effects of inflammatory cytokines are related to the expression of different molecules, intraglomerular hemodynamic abnormalities, alteration of extracellular matrix, and glomerular basement membranes, apoptosis and necrosis, endothelial permeability, oxidative stress, and so forth [21, 52–57], determining the development of microvascular diabetic complications, including neuropathy, retinopathy, and nephropathy [24, 49, 58–61].

Serum and urinary levels of interleukin- (IL-) 18 have been reported to be higher in patients with DN than in control subjects, showing significant positive correlations with UAE rate in DN patients [62–64]. IL-18 is a potent proinflammatory cytokine implicated in different actions, including the release of interferon- (IFN-) *γ* [65], which stimulates functional chemokine receptor expression in human mesangial cells [66], the synthesis of other molecules involved in the inflammatory reaction, such as IL-1 and TNF-*α*, the increase in the expression of ICAM-1, and the apoptotic process of endothelial cells [67–69]. Tubular renal cells show an increase in the expression of IL-18 in patients with DN [70], which has been related to the triggering of mitogen-activated protein kinase (MAPK) pathways secondary to the action of TGF-*β* [71]. Many other cells may also produce this cytokine, such as infiltrating monocytes, macrophages, and T cells [72, 73].

Renal cells (endothelial, epithelial, mesangial, and tubular cells) are also capable of synthesizing proinflammatory cytokines such as TNF-*α*, IL-1, and IL-6, and, therefore, these cytokines, acting in a paracrine or autocrine manner, may induce a variety of effects on different renal structures [51, 74, 75] playing a significant role in the development and progression of several renal disorders [51].

TNF-*α* is mainly produced by monocytes, macrophages, and T cells but also intrinsic kidney cells [46, 76, 77]. Many clinical studies in patients with DN have reported that the serum and urinary concentrations of TNF-*α* are elevated as compared with nondiabetic individuals or with diabetic subjects and kidneys and that these concentrations increase concomitantly with the progression of DN. These findings indicate a potential relationship between the elevated levels of this inflammatory cytokine and the development and progression of renal injury in DM [13, 62, 78]. Experimental studies in animal models of diabetes have showed that TNF-*α* protein and expression levels are enhanced in renal glomeruli and tubules [46, 79–81]. TNF-*α* may cause direct cytotoxicity to renal cells, inducing direct renal injury [82], apoptosis, and necrotic cell death [83, 84]. It can also produce alterations of intraglomerular blood flow and reduction of glomerular filtration as consequence of the disequilibrium between factors promoting vasoconstriction and vasodilation [85], in addition to changes in the permeability of endothelial cells. In addition, TNF-*α* is able to directly induce the formation of ROS by renal cells [56]. Experimental researches have shown that TNF-*α* induces the activation of NADPH oxidase in isolated rat glomeruli through the activation of the intracellular pathways protein kinase C/phosphatidylinositol-3 kinase and MAPK [56]. Thus, TNF-*α* prompts local ROS production, independent of hemodynamic mechanisms, resulting in alterations of the glomerular capillary wall and consequently increased albumin permeability [86].

Kidney hypertrophy and hyperfiltration are early and relevant findings of DN, and both are significantly related to TNF-*α* [81, 87].* In vitro* studies demonstrated that TNF-*α* stimulates the solute uptake in proximal tubular cells secondary to the activation of sodium-dependent cotransporters [88], whereas* in vivo* studies in diabetic rats found an enhanced urinary excretion of TNF-*α* excretion, which was related to sodium retention and renal hypertrophy. All these effects could be blocked by the use of a soluble TNF-*α* receptor fusion protein [81, 88]. In the renal distal tubule TNF-*α* activates the epithelial sodium channel resulting in an increased reabsorption of sodium, which can be abrogated by blockers of this renal channel, such as amiloride, and inhibitors of extracellular signal related protein kinase. The increment in renal sodium reabsorption might induce the expression of TFG-*β*, with the development of renal hypertrophy [89].

Similarly to TNF-*α*, IL-6 levels are also higher in patients with DN in comparison with diabetes mellitus patients without nephropathy [90]. In addition, the histopathological analysis of human renal samples by immunohistochemistry has demonstrated an increased expression of mRNA encoding IL-6 in cells infiltrating the mesangium, interstitium, and tubules, with a positive relationship with the severity of mesangial expansion [91]. Other functional and structural abnormalities related to DN and progression of renal damage have been associated with IL-6, including abnormalities in the permeability of glomerular endothelium, expansion of mesangial cells and enhanced expression of fibronectin [54], and increase in the thickness of the GBM [92, 93]. Our experimental studies have demonstrated an increase in the mRNA levels of IL-6 in the renal cortex of diabetic rats, which is positively associated with the urinary concentration of this cytokine [79]. In addition, in animal models of diabetes, wet kidney weight, a marker of renal hypertrophy and an early phenomenon in kidney involvement in DM, has been reported to be enhanced, which was related to mRNA gene expression levels and urine concentration of this cytokine [79].

## 4. New Therapies Targeting Inflammation

Nowadays, there are no available treatments to prevent the development of DN. Main therapeutic strategies are based on strict control of mayor modifiable risks like hypertension, glucose levels, and dyslipidemia but do not always prevent the ultimate progression of DN [94]. Therefore, the identification of therapies that specifically target DN by affecting the primary mechanisms that contribute to the pathogenesis could be useful and really needed in addition to risk factors control [95].

Inflammation process underlays the mechanisms of DN progression. Therefore, anti-inflammatory strategies may offer approaches of great interest in these patients. Several currently used treatments associated with renoprotective effects are postulated to be at least partly related to anti-inflammatory actions. The renin-angiotensin-aldosterone system (RAAS) is a major pathway involved in the pathogenesis and progression of DN [96]. Therapeutic RAAS blockade is achieved by two ways: by using angiotensin-converting enzyme (ACE) inhibitors or angiotensin II receptor (AR) blockers. Both are effective strategies that reduce proteinuria and slow progression of diabetic and nondiabetic nephropathy by hemodynamic/antihypertensive and by anti-inflammatory/antifibrotic actions. The second action is mediated by the reduction in angiotensin II (AngII) levels, which activates nuclear factor (NF-*κ*B) and interacts with transforming growth factor-*β* (TGF-*β*). The anti-inflammatory action occurs via inhibition of NF-*κ*B-dependent pathways [97].

Although the RAAS blockade provides pleiotropic, anti-inflammatory actions potentially relevant in the therapeutic approach to this complication [98–101], new therapeutic agents with potential effects upon primary mechanisms are on the horizon. One of these alternatives could be based on the use of pentoxifylline (PTF) which possesses significant anti-inflammatory properties. PTF is a methylxanthine-derived phosphodiesterase inhibitor with beneficial effects on microcirculatory blood flow due to its rheological properties. PTF is employed in the use of intermittent claudication resulting from peripheral vascular disease. In patients with DM, PTF therapy has been associated with a reduction in UAE and with potential beneficial effects on GFR [102–106]. Recent studies have shown that PTF reduces urinary protein excretion in diabetic subjects, both with normal renal function [107, 108] and with renal insufficiency [106]. Interestingly, this antiproteinuric effect has been related to a reduction in the concentrations of TNF-*α*, one of the most important proinflammatory cytokines [106, 109]. This antiproteinuric action has been confirmed in various prospective, controlled, randomised clinical studies [108, 110, 111]. The drug inhibits TNF-*α* gene transcription and blocks TNF-*α* mRNA accumulation [100, 112] significantly reducing TNF-*α* levels and urinary protein excretion without metabolic or haemodynamic changes [106–108], even in patients under blockade of the RAS with Ang II receptor antagonists [109]. These studies showed a significant association between the reduction in proteinuria and the decrease in TNF-*α* activity [106, 109]. In addition, PTF has a considerable capacity to modulate other proinflammatory cytokines and molecules, including IFN-*γ*, IL-10, and IL-6, as well as to attenuate cellular processes involved in the inflammatory response (activation, adhesion, and phagocytosis) without metabolic or haemodynamic changes [113–115]. A meta-analysis published in 2008 which focused on the use of PTF in patients with DN found a substantial reduction in urinary protein excretion and pointed to the capacity of PTF to reduce the production of proinflammatory cytokines as the most likely explanation for this antiproteinuric action [116]. Therefore, PTF could represent a therapeutic approximation to the anti-inflammatory treatment of DN.

One* in vitro* study has showed that PTF decreased cellular production of fibronectin and TGF-*β* induced by high glucose concentrations in cultured human mesangial cells and exerted protective effects against angiotensin-II-induced actions on matrix proteins [117]. Recent experimental studies in animal diabetic models show that administration of PTF prevents an increase in renal expression, synthesis, and excretion of TNF-*α*, IL-1, and IL-6, which was directly and significantly associated with a reduction in renal sodium retention, renal hypertrophy, and urinary albumin excretion [79, 109].

An independent, prospective, randomized, controlled, clinical trial investigating the potential renoprotective effect of PTF administration in patients with DN, under standard care with RAS blockers, recently reported a slowing of the rate of progression of nephropathy among patients with type 2 diabetes [118] with a smaller decrease in eGFR and a higher reduction of residual UAE compared with control group nontreated with PTF. Patients showed a reduction in urinary TNF-*α* after PTF administration, which was directly correlated with the change in UAE and inversely correlated with the variation in the eGFR. No significant relationship was observed between serum and urinary levels of this cytokine, indicating that TNF-*α* is produced within the kidneys and that PTF administration is associated with a modulation in its production and urinary excretion.

Further convincing evidence is, however, needed before pentoxifylline can be considered a real option for the treatment of DN. Therefore, PTF should not be considered part of clinical practice without more definitive trials (large-scale, adequately powered, multicenter, prospective, placebo-controlled studies, with definitive endpoints on efficacy and safety) to demonstrate with the maximum grade of evidence the renoprotective, anti-inflammatory properties of PTF in this population.

## 5. Conclusions

Providing diabetic patients with protection from the development and progression of renal injury remains a challenge for nephrologists. In this context, the need to identify new therapeutic targets and additional strategies for treating DN is clearly evident, especially since current treatments do not completely stop the development and progression of renal injury in the diabetic patient. Diabetic nephropathy is considered an inflammatory disease, and several reports recently demonstrated inflammasome activation in association with diabetic nephropathy [119]. The modulation of inflammatory processes might be useful in the prevention or therapy of DN. Inflammatory cytokines exert an important diversity of actions implicated in this disease, from development to the initial stages of diabetes to progression and to late stages of renal failure. The recognition of these molecules as significant pathogenic factors and the development of new techniques for examining changes in the expression of pathogenic genes involved in inflammatory pathways in this complication will provide new therapeutic targets.

From a therapeutic perspective, limited experience is available regarding the inhibition of inflammatory cytokines in DN. Mounting evidence implies beneficial properties of ACE inhibitors beyond those of their original effects. Therefore, modulation of inflammatory phenomena by blocking the RAS in DN is of great interest. Diverse* in vitro* and* in vivo* studies have shown that ACE inhibitors have inhibitory effects on proinflammatory cytokine expression and synthesis [79, 120–124] which are not related to the antihypertensive effects of these drugs [125]. Therapies with ACE inhibitors in patients with congestive heart failure or advanced chronic renal disease have demonstrated that is associated with a significant decrease in TNF-*α* and IL-6 activity [126, 127]. Based on these findings, it is possible to hypothesize that other angiotensin-dependent processes, such as those related to proinflammatory cytokine regulation, play a significant role in the development and progression of DN, and, therefore, blockade of cytokine-mediated inflammatory activity may have important effects on the renoprotective benefit associated with RAS blockade.

To date, diverse studies have shown that PTF administration is able to reduce the main proinflammatory cytokines related to a decrease in renal hypertrophy and UAE. These beneficial effects are independent of any improvement in metabolic or haemodynamic parameters [79]. However, further clinical trials are necessary to examine the potential renoprotective efficacy of PTF and other anti-inflammatory cytokines in establishing remission or even regression of DN.
